# A bibliometric analysis of global research on toxoplasmosis in the Web of Science

**DOI:** 10.14202/vetworld.2018.1409-1415

**Published:** 2018-10-12

**Authors:** Mahdi Fakhar, Masoud Soosaraei, Ali Akbar Khasseh, Reza Zolfaghari Emameh, Hajar Ziaei Hezarjaribi

**Affiliations:** 1 Molecular and Cell Biology Research Center, Department of Parasitology, Mazandaran University of Medical Sciences, Sari, Iran; 2 Student Research Committee, Department of Parasitology, Mazandaran University of Medical Sciences, Sari, Iran; 3 Department of Knowledge and Information Sciences, Payame Noor University, Tehran, Iran; 4 Department of Energy and Environmental Biotechnology, National Institute of Genetic Engineering and Biotechnology (NIGEB), Tehran, Iran

**Keywords:** bibliometric, scientific collaboration, *Toxoplasma* spp, toxoplasmosis, Web of Science

## Abstract

**Aim:**

This study was designed to evaluate the network productions and research collaborations on toxoplasmosis worldwide.

**Materials and Methods:**

A bibliometric research was carried out using the Web of Science (WOS) database. The analysis unit was the original research articles about toxoplasmosis published between 2000 and 2016 (17 years).

**Results:**

Totally, 6,550 articles about toxoplasmosis were indexed in the WOS with the following information: (A) 18,410 researchers played a role in drafting the articles; (B) 33 different countries have contributed in the toxoplasmosis studies; (C) the USA was ranked at the first place with 2,162 publications about toxoplasmosis; and (D) “Dubey JP” was compiled and participated in 401 articles from the USA, as the highest number and main core of publications in the toxoplasmosis network.

**Conclusion:**

The main focus of the toxoplasmosis research activities in the world was article production in the indexed journals in WOS. Hence, it is necessary to strengthen the collaboration networks to improve the quality of articles. Furthermore, the priority would be the identification of institutions with a higher number of research article productions in WOS, to perform toxoplasmosis collaborative original researches according to the strategic roadmap and scientific plan of each country.

## Introduction

Toxoplasmosis is caused by the protozoan *Toxoplasma gondii*. This parasite is a cosmopolitan protozoan classified as a coccidian in the phylum Apicomplexa. As a zoonotic agent, *T. gondii* is an obligate intracellular parasite in both humans and animals [1]. *T. gondii* is the causative parasite of both congenital infection and abortion in human and livestock [2]*. T. gondii* infects most genera of warm-blooded animals, but the primary host is the family Felidae. The infection occurs through ingestion of either contaminated food, water, or dust [3,4]. Furthermore, the parasite can be found as tissue cyst in all livestock. Ingestion of raw or undercooked infected meat is the second etiological mode of transmission. The infection transmission, for example, through infected transplants or vertically *in utero*, is an additional route of transmission [5,6]. The bibliometric analysis is a statistical approach used to assess the quantity and quality of publications, such as books or peer-reviewed articles on a certain topic [2]. In recent years, there have been growing international bibliometric studies in different fields of medicine [7-9]. The bibliometric analysis on toxoplasmosis literature will give us an idea about this subject and the content of the articles has been most cited. Furthermore, it will shed light on the network of authors and co-authorship and find research partners worldwide for potential collaboration and joint grant seeking. In addition, the scientometric analysis is an important indicator of the impact of governmental and non-governmental initiatives on the parasitic disease [2,3]. Therefore, according to the objective of this study, we are going to search toxoplasmosis-related literature using Web of Science (WOS) as an online subscription-based scientific citation indexing service. Through this analysis, a series of criteria such as the number of publications, highly active authors, countries, and institutions, top-cited articles, international collaborations, and the target journals for publication of the articles on toxoplasmosis will be evaluated. Thus, the estimation of global and regional productivity of ongoing research on this cosmopolitan infection may be of interest.

The bibliometric analyses are generally focused on the study of scientific production, citation, or impact factor of the journals in the field. However, there are no comprehensive data related to the production of toxoplasmosis field.

The collaboration among authors and the formation of research groups in the field have not been evaluated. Furthermore, no study has performed to evaluate whether the research collaboration is a primary study on the same or different strains of *Toxoplasma* spp. (different interests would be existed to obtain the results for the control and management of the disease). In this sense, the study of scientific co-authorship through social network analysis advises us to analyze the precise structure of the collaboration within a discipline or area of knowledge than to study based on the bibliometric indicators alone.

The collaboration of researchers in production of toxoplasmosis articles from different countries, regardless of the different forms of the infection, can be considered as a critical factor in the formation and evolution of research groups in the field.

Since the present study is a documented scientometric production, the main purpose would be the study of articles regarding toxoplasmosis topic published between 2000 and 2016 (17 years) in the WOS database.

To achieve the aims, the answering to the following questions is needed:

- How have you included in the international research articles on toxoplasmosis in the journals from Institute for Scientific Information (ISI)?
- Who are involved in the study of toxoplasmosis in terms of number of publications, citations, and Hirsch index (*h*-index)?
- Which countries have performed most of the studies on toxoplasmosis and what is the geographical distribution of the leading countries in the study?
- What is the frequency of the keywords used in the title and summary of the papers in the article-based analysis?
- What kind of keywords are used in the bibliometric analysis of toxoplasmosis?

Given how important research on toxoplasmosis is to global health, it is necessary to prepare a comprehensive view of the status of this study in the world and a clear picture of the production process and scientific exchanges in the field. This study will also inform us about the interests of the scientists on each aspect of toxoplasmosis. Noticeably, the improvement of the scientific situation in the field of toxoplasmosis will lead to progress in prevention, treatment, and reduction of complications.

Thus, the aim of the present study was the bibliometric analysis of the global scientific publications and identification of the top researchers in the field of toxoplasmosis and their geographic distribution.

## Materials and Methods

### Ethical approval

This study was not based on live animals.

### Type of study

This research is a scientometrics publication in the field of toxoplasmosis including published articles in the WOS database throughout 2000-2016, which are the products were indexed in the ISI database.

### Search strategy

Titles, abstracts, and keywords were searched using the shortened word “*Toxoplasma* spp.” or “toxoplasmosis.” The search results were filtered by document types including research and review articles as well as proceeding papers, which were indexed in Social Science Citation Index, Conference Proceedings Citation Index-Science, Emerging Sources Citation Index, Science Citation Index-expanded, and Conference Proceedings Citation Index-Social Science, and Humanities (timespan between 2000 and 2016).

## Results

### General data

**Figure-1:**
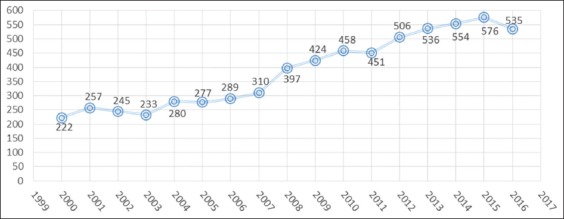
Quantitative growth process of the *Toxoplasma* studies during 17 years passed from the beginning of the 21^st^ century.

The results of the study showed 6,550 articles on *Toxoplasma* spp. between 2000 and 2016, which all were indexed in the WOS. Furthermore, results indicated that international research on *Toxoplasma* has grown steadily within 21^th^ century. In other words, the number of *Toxoplasma* articles ranged from 222 in 2000, to 535 in 2016 (Figure-1).

### Analysis of publications by journal

**Figure-2:**
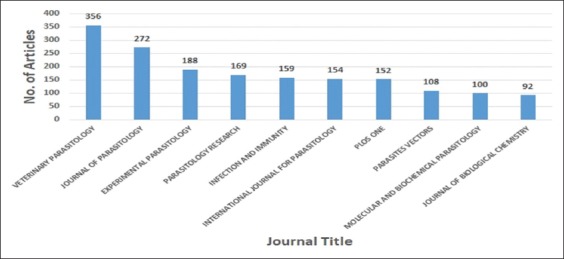
Top ten journals have published articles about *Toxoplasma* between 2000 and 2016.

The most important journals usually contain articles with the highest impact in the studied area. Thus, subscriptions to such journals in indexing and abstracting services would be justified scientifically. Most of the top journals publishing on toxoplasmosis were from the parasitology and veterinary subject categories. The top journal was Veterinary Parasitology, while the Journal of Parasitology was ranked in the second journals (Figure-2).

### The researcher with the highest number of studies

Through reviewing and assessing of various indices for *Toxoplasma* researchers, it was revealed that the most important of them could be marked based on the index number and citation of their articles. Among these indicators, the index number is already used in many universities in Iran. Preliminary analysis of the obtained data showed that a total of 18,410 researchers played the major role in publication of the 6,550 articles, in which “Dubey JP” played an active role through compiling and participation in publication of 401 articles about *Toxoplasma* in the WOS, while this record had a great difference with other researchers of *Toxoplasma*. “Zhu XQ” and “Sibley LD” with 153 and 112 articles have been ranked in the second and third places, respectively (Table-1).

**Table-1:** The highly cited researchers in the field of *Toxoplasma* studies.

Rank	Researcher name	Number of articles
1	Dubey JP	401
2	Zhu XQ	153
3	Sibley LD	112
4	Boothroyd JC	96
5	Gennari SM	92
6	Kwok Och	85
7	Mineo JR	75
8	Villena I	73
9	Liesenfeld O	72
10	Roos DS	69

The productivity of researchers can be measured by the number of articles they publish, but what is clear is that such an index will be incomplete, because the ideas contained in these studies should be studied and cited by other researchers. Thus, the influence of researchers is associated both by a number of publications and number of citations. Furthermore, as the first ranked scientist, “Dubey JP” received 11,260 by a wide margin, according to citation by other researchers working on *Toxoplasma* in the ISI and “Sibley LD” and “Boothroyd JC” got 6,696 and 5,854 citations and stood in the second and third place, respectively (Table-2).

**Table-2:** The most cited researchers in the field of *Toxoplasma* studies.

Rank	Researcher name	Number of citations
1	Dubey JP	11,260
2	Sibley LD	6,696
3	Boothroyd JC	5,854
4	Roos DS	3,278
5	Su C	3,119
6	Liesenfeld O	2,888
7	Darde Ml	2,571
8	Jones Jl	2,514
9	Dubremetz JF	2,480
10	Kwok Och	2,457

In addition, *h*-index was applied for identification of the most influential and productive researchers in the field of *Toxoplasma* studies. This study was designed to show the cumulative effect of the research output of the researcher. *H*-index has the potential to cite two important indicators of the number of articles and simultaneously prepare a framework to assess the influence of a researcher.

As it is shown in Table-3, “Dubey JP” with *h*-index=53 has the highest score among all researchers working on *Toxoplasma*.The *H*-index=53 means that “Dubey JP,” has published at least 53 papers that have each been cited at least 53 times. In this respect, “Sibley LD” and “Boothroyd JC” with *h*-index=52 and 41 were ranked in the second and third places, respectively.

**Table-3:** Top researchers in the field of *Toxoplasma* based on *h*-index.

Rank	Researcher names	number of articles	Number of citations	*h*-index
1	Dubey JP	401	11,260	53
2	Sibley LD	112	6,696	52
3	Boothroyd JC	96	5,854	41
4	Roos DS	69	3,278	35
5	Su C	66	3,119	31
6	Kwok Och	85	2,457	29
7	Carruthers VB	66	2401	29
8	Petersen E	65	2317	29
9	Thulliez P	49	2,135	28
10	Hunter Ca	58	1,922	28

As an interesting point in Table-3, both of “Roos DS” and “Su C” have published more articles than “Kwok Och,” while *h*-index for “Kwok Och” was higher than the other two. It seems that “Kwok Och” has performed the impressive works compared to other two researchers. It is also interesting to note that this article contains 112 “Sibley LD” with *h*-index=52, while “Dubey JP” has far more articles (401 articles) with *h*-index=53, which looks that the research performed by “Sibley LD” had a higher impact.

### Contribution of journals in the publication

During the 17-year (2000 – 2016), *Toxoplasma* studies were published in various journals. The journal of “Veterinary Parasitology” hosted 356 articles, which played the great contribution in the expansion and development of *Toxoplasma* studies. The journals of “Parasitology” and “Experimental Parasitology” were ranked in the second and third places with publication of 272 articles and 188 articles, respectively. Furthermore, 1,750 articles were published in ten journals. It means that 72.26% of the articles on *Toxoplasma* have been published in these journals.

### Geographical distribution

**Figure-3:**
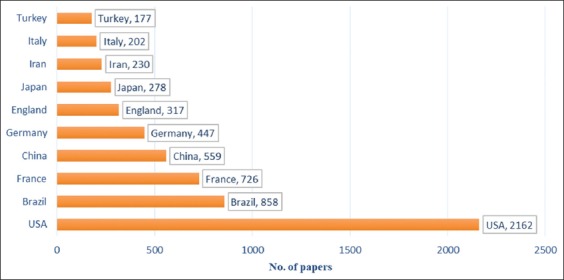
Top ten countries producing articles in the field of *Toxoplasma* study.

**Figure-4:**
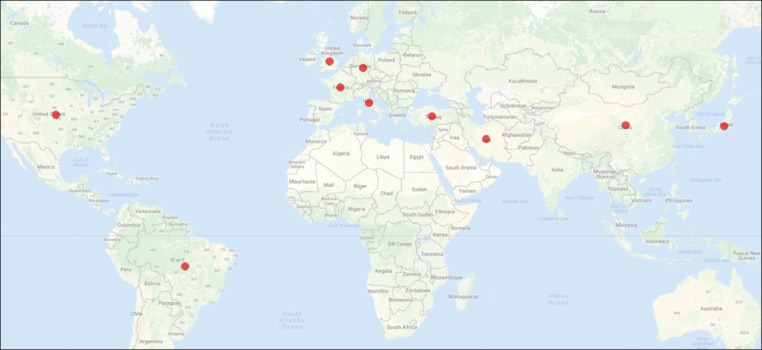
Geographical dispersion of top ten leading countries in the studies of *Toxoplasma*.

After rating the countries participating in the study of toxoplasmosis, it was revealed that the United States by 2,162 articles was ranked in the first place during 2000-2016. Furthermore, the researchers from 33% of the world countries have contributed to *Toxoplasma* study (Figures-3 and 4). In addition, the USA was the leading country in the publication on toxoplasmosis, while the cases of this parasitic infection were less than South America. Brazil, a country with a high prevalence of toxoplasmosis, led scientific production on leishmaniasis in Latin America. This can be attributed to the number of researchers and development of the country’s scientific system, which has become the principal scientific reference for South America [9-11]. France, as a European country and high prevalence of toxoplasmosis [12], was ranked as the third country in the production of toxoplasmosis-related articles.

### Map of Toxoplasma studies by frequency of the title words

**Figure-5:**
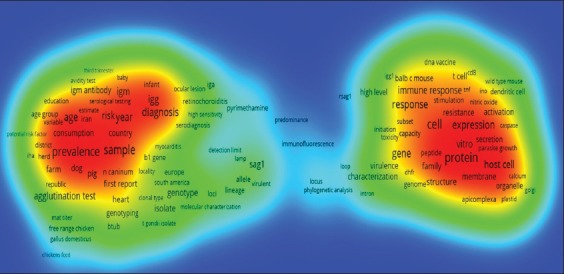
Density view of the most frequent terms used in world studies of *Toxoplasma*.

Analysis of toxoplasmosis studies based on the frequency of the words used in the title and summary of the articles showed the interest, emphasize, and ability of researchers disclosing certain words and topics. This enabled us to acquire conceptual visual and overview of the content of this study. Figure-5 shows the density of frequent words used in the title and abstract of *Toxoplasma* studies. Red dots indicate the density of the *Toxoplasma* studies compared to other parts of the world. Furthermore, the words such as “protein,” “cell,” “sample,” and “prevalence” are reflecting the higher frequency of *T. gondii* in the studies in comparison to other words represent the bigger letter size (Figure-5). Table-4 shows the most frequent words used in the *Toxoplasma* study. As Table-4 shows, the words “protein,” “cell,” and “sample” with 1,012, 989, and 912 repeats in the title or summary of the articles on *Toxoplasma* studies have been ranked in first, second, and third places, respectively.

**Table-4:** The most frequent words used in the *Toxoplasma* studies.

Rank	Phrase	Frequency
1	Protein	1,012
2	Cell	989
3	Sample	912
4	Prevalence	895
5	Seroprevalence	783
6	Gene	672
7	Response	660
8	Diagnosis	650
9	Year	648
10	Age	646
11	Cat	638
12	Mechanism	626
13	Activity	624
14	Expression	599
15	Serum sample	571

### Analysis of keywords using the word cloud tool

**Figure-6:**
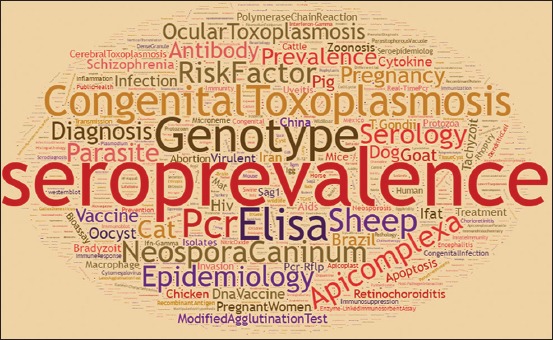
Word cloud of global research keywords of *Toxoplasma*.

For this purpose, after the detection of keywords in *Toxoplasma* studies and using the following online service: (https://timdream.org/wordcloud/) as a visual semantic network, the word cloud image was drawn (Figure-6). It should be noted that the size of the image indicates the frequency of keywords in the articles. For example, keywords such as seroprevalence, ELISA, and congenital toxoplasmosis were drawn using larger words which indicate the highest frequency of the keywords in the study of *Toxoplasma*. It should be noted that, in this part of the study, three words including toxoplasmosis, *Toxoplasma* spp., and *T. gondii* have been repeated with the highest frequency among other keywords.

## Discussion

The global incidence and burden of disability-adjusted life years of congenital toxoplasmosis are annually estimated 190,100 cases and 1.2 million with high burden in South American countries such as Brazil [13-15]. Furthermore, the seroprevalences in childbearing age women were ranged between 4% and 85% (Table-3). Only a small percentage of toxoplasmosis in the adult population was acquired vertically. Not only all possible routes of infection are important epidemiologically, but also all sources of infection may vary greatly among different ethnic groups and geographical locations. Therefore, knowing more about the probable routes of horizontal transmission of infection to human and the most likely sources of infection in a given population are the pre-requisites of the development of effective strategies for the prevention of infection in the risk groups, such as non-immune pregnant women and immunocompromised patients, in particular, those with acquired immunodeficiency syndrome (AIDS) [16-18]. In this study, a bibliometric study was performed on the toxoplasmosis publications, which has indicated an increase in the number of publications on toxoplasmosis over 17 years between 2000 and 2016. Since no work has been done on the bibliometric aspects of toxoplasmosis, this study can define the present status of studies on toxoplasmosis in the world.

Moreover, concerns about the lack of well-controlled studies, potential costs, and harms of the programs outweigh benefits in a practical setting. In addition, in some countries, obstetricians and gynecologists have not recommended universal screening of *Toxoplasma* infections in pregnant women in their practice guidelines [1]. On the other hand, there are some studies and systematic reviews showing the conflicting results about prenatal screening and treatment of congenital toxoplasmosis [18-22]. There is a necessity for well-controlled studies to determine the effectiveness of these interventions. Our study showed that the number of publications on toxoplasmosis oscillated in the past decade. However, referring to the number of toxoplasmosis publications within the past five decades, it was obvious that there was an overall increase in the number of publications in the past 17 years.

## Conclusion

We estimated the research productivity in the field of toxoplasmosis worldwide for 17 years. Our study provided informative data that may be used by funding agencies and governmental bodies regarding the development of research networks between developing and developed countries. It seems that these kinds of studies can be led to deeper biomedical research collaboration in the field of toxoplasmosis through identification of high-ranked researchers from both developing and developed countries.

## Authors’ Contributions

MF, MS and AAK designed all steps of the study, HZH and RZE reviewed the manuscript, and MS wrote the manuscript draft. All authors read, revised, and approved the final manuscript draft.
